# The complete genome sequence of *Paludicola* sp. MB14-C6, isolated from the lake waters of Donghu, Wuhan City, China

**DOI:** 10.1128/mra.01100-23

**Published:** 2024-05-29

**Authors:** Jingjing Zhao, Chao-Jen Shih, Yen-Chi Wu, Shu-Jung Lai, Yi-Ting You, Sheng-Chung Chen

**Affiliations:** 1 School of Resources and Chemical Engineering, Sanming University, Sanming, Fujian, China; 2 Fujian Provincial Key Laboratory of Resources and Environmental Monitoring and Sustainable Management and Utilization, Sanming University, Sanming, Fujian, China; 3 Bioresource Collection and Research Center, Food Industry Research and Development Institute, Hsinchu, Taiwan; 4 Graduate Institute of Biomedical Sciences, China Medical University, Taichung, Taiwan; 5 Research Center for Cancer Biology, China Medical University, Taichung, Taiwan; 6 Department of Life Sciences, National Chung Hsing University, Taichung, Taiwan; SUNY College of Environmental Science and Forestry, Syracuse, New York, USA

**Keywords:** *Paludicola*, Donghu lake

## Abstract

Here, we report the complete genome sequence of *Paludicola* sp. strain MB14-C6, which was isolated from the lake waters of Donghu, situated at Wuhan City, Hubei Province, China. The genome of strain MB14-C6 was chosen for further species delineation and comparative genomic analysis.

## ANNOUNCEMENT

In this study, the culture-dependent method has been used to investigate prokaryote diversity in Donghu in Wuhan. Strain MB14-C6, one of our purified isolates, may represent a new species, prompting its selection for genome sequencing to analyze species delineation. Water sample from Donghu was collected on 3 May 2021 and subsequently inoculated into the modified anaerobic DSM924 medium [per liter: 1 g MgCl_2_ × 7H_2_O, 0.5 g KCl, 0.4 g CaCl_2_ × 2H_2_O, 0.4 g K_2_HPO_4_, 1 g NH_4_Cl, 10 mL trace element solution, 2 g yeast extract, 2 g tryptone, 0.5 mL Na-resazurin solution (0.1%), 4 g NaHCO_3_, vitamin solution, 0.25 g L-cysteine-HCl × H_2_O, 0.25 g Na_2_S × 9H_2_O, and headspace: N_2_:CO_2_ = 4:1]. The culture was incubated at room temperature (~25°C) for 2 weeks, and then strain MB14-C6 was purified using the roll-tube technique, a method employed for isolating strict anaerobes from a thin layer of agar medium covering the internal surface of anaerobic tubes (1). The primers of 8F (5′-AGAGTTTGATCCTGGCTCAG-3′) and 1492RU (5′-TTTTAATTAAGGTTACCTTGTTACGACTT-3′) were used for 16S rRNA gene amplification (2). According to the analysis of the 16S rRNA gene using EzBioCloud’s identification service (3), the result indicates that strain MB14-C6 was most closely related to *Paludicola psychrotolerans* (4, 5) with 95.62% similarity, leading to the conclusion that strain MB14-C6 belongs to the genus *Paludicola*.

The strain was cultivated in 1 L of modified DSM924 medium at 30°C for 7 days, and genomic DNA extraction from the resulting cell pellet was conducted using the NucleoBond HMW DNA kit (Macherey-Nagel, Germany) following the manufacturer’s guidelines. Whole genome shotgun sequencing was carried out utilizing the DNBSEQ-T7 platform (MGI Tech Co., Ltd.) and the MinION sequencer (Oxford Nanopore Technology) at the Sangon Biotech (Shanghai) Co., Ltd.

For the DNBSEQ-T7 platform, DNA fragments sheared to approximately 300 bp using the Covaris sonicator were utilized to prepare a 150 bp paired-end DNA library, which employed the Hieff NGS MaxUp II DNA Library Prep Kit for Illumina (Yeasen Biotechnology, Shanghai, Co., Ltd.). The resulting library was sequenced using MGISEQ-2000RS High-throughput Sequencing Set, yielding 13,019,004 reads that underwent trimming via Trimmomatic v0.36 (6). For the MinION sequencer, DNA fragments underwent end-repair, 3’ adenylation, and ligation to adaptors pre-loaded with motor proteins. Subsequently, fragments larger than 1 Kb, purified with Agencourt AMPure XP Beads (Beckman, A63881), underwent single-molecule nanopore DNA sequencing using Multiplex Ligation Sequencing Kit (SQK-MLK111.96-XL) on MinION Flow Cell (R9.4.1). Basecalling was executed with Guppy version 6.5.6 using the default model [Oxford Nanopore Technology (ONT)], resulting in 506,850 reads (length-bp: mean = 3,711.5; median = 2,099; longest = 126,103). Trimming was performed with Porechop v0.2.4 (7), and filtering was carried out with NanoFilt v2.8.0 (8). The DNBSEQ-T7 and MinION reads were then hybrid *de novo* assembled using Canu v2.2 (9), yielding an assembly *N*_50_ value of 2,937,905 bp. The hybrid sequencing approach provided approximately 939× mean coverage of the genome. Gene predictions and annotations were carried out using NCBI Prokaryotic Genome Annotation Pipeline v6.5 (10).

The assembly generated a single large contig of 2,937,905 bp with 34.1% GC content. The circularization was autonomously performed by the Canu software without rotation and predicted 2,793 genes. Default parameters were applied for all bioinformatic analyses.

## Data Availability

The genome sequence of strain MB14-C6 has been deposited in GenBank under accession number CP133133. The version of the genome described in this paper is first version. The BioProject and BioSample accession numbers are PRJNA1005944 and SAMN37004496. Raw reads obtained from DNBSEQ-T7 and MinION were deposited in the Sequence Read Archive (SRA) under accession numbers SRR26730975 and SRR26730976, respectively.
